# Non-alcoholic fatty liver disease and correlation of serum alanin aminotransferase level with histopathologic findings

**Published:** 2011-06-01

**Authors:** Somaye Khosravi, Seyed Moayed Alavian, Ali Zare, Nasser Ebrahimi Daryani, Seyed-Mohammad Fereshtehnejad, Narges Ebrahimi Daryani, Mohammad Reza Keramati, Sina Abdollahzade, Sahar Taba taba Vakili

**Affiliations:** 1 Department of Gastroenterology, Tehran University of Medical Sciences, Tehran, IR Iran; 2 Baqiatallah Research Center for Gastroenterology and Liver Diseases, Tehran, IR Iran; 3 Department of Pathology, Tehran University of Medical Sciences, Tehran, IR Iran; 4 Gastrointestinal & Liver Disease Research Center (GILDRC), Firoozgar Hospital, Tehran University of Medical Sciences, Tehran, IR Iran; 5 Department of Gastroenterology, Shahid Beheshti University of Medical Sciences, Tehran, IR Iran

**Keywords:** Non-alcoholic fatty liver disease, Serum alanine aminotranferase, Histopathology

## Abstract

**Background:**

Non-alcoholic fatty liver disease (NAFLD) has been recognized as the most common cause of chronic liver disease worldwide. It occurs in patients who do not consume alcohol in large amounts. Alanine aminotranferase (ALT) and aspartate aminotransferase (AST) are indicators of hepatocellular injury.

**Objectives:**

To determine correlation between histopathologic specifications of NAFLD in patients with little or no history of alcohol consumption and the serum level of ALT.

**Patients and Methods:**

In a cross-sectional study carried out in two gastroenterology and hepatology clinics in Tehran, Iran, the medical records of those who had undergone liver biopsies between years 2005 and 2009 were reviewed. Clinical and laboratory information of biopsy-proven cases of NAFLD were obtained from 147 eligible medical records. The histopathologic, demographic, and laboratory data of the participants were also collected. Two groups of patients according to their serum ALT level (cut-point of 35 U/L) were defined. The quantitative pathologic grade of the biopsy specimens was determined based on Brunt scoring system.

**Results:**

We studied 147 NAFLD patients including 127 men (86.4%) and 20 women (13.6%) with a mean ± SD age of 41.4 ± 11.2 years. Considering serum ALT, the mean ± SD quantitative grade of hepatosteatosis was 1.50 ± 0.67 and 1.74 ± 0.73 (p=0.136); advanced fibrosis (consisted of grade III and cirrhosis) was found in 4.5% (1/22) and 5.6% (7/125) of patients (p=0.327).

**Conclusions:**

We found that using the cut-off value of 35 U/L for serum ALT level, it has little contribution to predict NAFLD severity.

## 1. Background

Non-alcoholic fatty liver disease (NAFLD) has been recognized as the most common cause of chronic liver disease worldwide [1][2][3][4]. By definition, NAFLD occurs in patients who do not consume large amount of alcohol. NAFLD has a wide histological spectrum ranging from macrovesicular steatosis which is usually a benign and non-progressive process to non-alcoholic steatohepatitis (NASH), liver cirrhosis, portal hypertension and hepatocellular carcinoma [5][6][7][8].

NAFLD is frequently associated with obesity, diabetes mellitus and the metabolic syndrome. Currently, it is considered as a part of the metabolic syndrome [9][10]. Hepatic transaminases, alanine (ALT) and aspartate aminotransferase (AST) are indicators of hepatocellular injury. Several studies demonstrated that high levels of ALT are correlated with a higher risk of NASH[11][12]. However, some studies have shown that patients with normal ALT levels may also have histological features of NASH and be at risk for disease progression [13][14]. Additionally, some recent studies have introduced a new ALT upper limit for a healthy individual which is ≤ 40 U/L for both genders. Elevated levels of ALT are sometimes associated with underlying NAFLD [15]. On the other hand, the current evidence on this issue is inconclusive and different normal ranges have been proposed.

## 2. Objectives

We conducted this study to determine clinical and histopathologic characteristics of patients with NAFLD and their possible correlation with serum ALT level.

## 3. Patients and Methods

This cross-sectional study was carried out in two gastroenterology and hepatology clinics in Tehran, Iran. Considering all medical records, consecutive biopsy-proven cases of NAFLD were recruited. Initially, the medical records of those who had undergone liver biopsy between 2005 and 2009 were reviewed and those with the following criteria were selected: Confirmed diagnosis of NAFLD with clinical criteria and biopsy specimens. Only those liver biopsy specimens were considered which represented fatty liver disease in case of predominantly macrovesicular steatosis or documented steatohepatitis. Steatohepatitis was also defined by the minimal criteria of hepatic steatosis and scattered, mainly lobular inflammation with or without Mallory bodies, cytologic ballooning, and perisinusoidal fibrosis. Another inclusion criteria was negative serologic markers of viral or autoimmune hepatitis (i.e., HBsAg, HCV Ab [ELISA], HIV Ab [ELISA], antinuclear antibodies, anti-smooth muscle antibodies, anti-liver/kidney microsomes type 1 antibodies and negative alpha-1 antitrypsin).

NASH is most often discovered during routine laboratory testing. Additional tests help to confirm the presence of NASH and rule out other types of liver disease. Imaging modalities (e.g., CT scan, or magnetic resonance imaging) may depict fat accumulation in the liver but cannot help in differentiation of NASH from other causes of liver disease that have a similar picture. A liver biopsy is required to confirm NASH. Blood tests to measure the liver function, levels of substances produced or metabolized by the liver can also help to diagnose NASH and differentiate NASH from alcoholic hepatitis. Levels of two liver enzymes (AST and ALT) are elevated in about 90% of people with NASH.

Moreover, additional blood tests are useful for ruling out other causes of liver disease. These usually include tests for viral hepatitis (hepatitis A, B, or C), and may include tests for less common causes of liver disease. Hemochromatosis and Wilson disease were ruled out by measuring serum levels of iron, and copper as well as ceruloplasmin, respectively. In addition, since patients who had drunk more than 20 g of alcohol per week, was more likely to suffer from other liver disorders including Wilson disease, hemochromatosis, primary biliary cirrhosis, primary sclerosing cholangitis, and biliary obstruction, and those who consumed drugs known to cause steatohepatitis (e.g., glucocorticoids, synthetic estrogens, aspirin, tamoxifen, amiodarone, calcium-channel blockers, and methotrexate), and patients who had positive history of jejunoileal bypass surgery, pregnancy or incomplete medical records were all excluded from the study.

The study protocol was approved by Ethical Committee of Tehran University of Medical Sciences.

### 3.1. Clinical and Laboratory Data

Clinical and laboratory information were obtained from 147 eligible medical records. Also, demographic and baseline data (patients' age , gender and body mass index [BMI]), laboratory information on levels of ALT and AST, alkaline phosphatase (Alk-P), total serum cholesterol and triglycerides, low density lipoprotein (LDL), high density lipoprotein (HDL), fasting blood sugar (FBS), partial thromboplastin time (PTT), serum platelet count and serum level of gamma-glutamyl transpeptidase (g-GTP) were recorded. These laboratory indices were measured using standard techniques. ALT activity was classified into "normal" and "elevated" according to the reference laboratory cut-off value of 35 U/L. In an attempt to determine the healthy range of ALT in the population under study, after excluding participants with positive viral markers, regular drug users, and alcohol drinkers, the upper normal limit (the 95th percentile) of serum ALT was 35 U/L. Thus, 22 patients with NAFLD had normal ALT (< 35 U/L), while the remaining 125 patients had elevated ALT (> 35 U/L). All the measured baseline, clinical and laboratory findings were compared between these two groups.

### 3.2. Histopathologic Assessment

All patients suspected to have NAFLD who showed no improvement in liver function after several months of diet and exercise therapy underwent liver biopsy. To document the definite diagnosis of NAFLD, all liver biopsy specimens were re-examined for fibrosis, steatosis, hepatocyte ballooning, and lobar and portal inflammation. The grading and staging of all biopsy specimens were done based on the method proposed by Brunt et al. by one expert pathologist [16]. Categorization of histopathologic findings by Brunt's method is presented in Table 1. The extent of steatosis was semiquantitatively graded from I to III considering percentage of cells with fatty droplets as follows: grade I, up to 33% steatosis; grade II, 33% to 66% steatosis; and grade III, > 66% of hepatocytes involved. Peri-sinusoidal fibrosis was scored from 0 to III based on the space of zone 3 region involved. The presence of cirrhosis was also noted. Moreover, "advanced fibrosis" was defined as the presence of bridging fibrosis (grade III) or cirrhosis.

All histopathologic features of the enrolled patients with NAFLD were also evaluated and compared between two groups with normal and elevated ALT. Patients with advanced fibrosis were omitted from parts of analysis because low level of serum ALT in this group of patients could hypothetically be attributed to the absence of enzyme-producing hepatocytes.

**Table 1:** Histopathologic grading of different findings in liver biopsies of patients with NASH

**Finding **	**Grading**
**Hepatosteatosis**	
**Grade I**	< 33% of hepatocytes affected
**Grade II **	33%–66% of hepatocytes affected
**Grade III**	> 66% of hepatocytes affected
**Fibrosis **	
**No fibrosis**	No fibrosis
**Grade I**	Zone 3 peri-sinusuidal peri-cellular fibrosis, focally or extensively present
**Grade II**	Zone 3 peri-sinusuidal peri-cellular fibrosis, with focal or extensive peri-portal fibrosis
**Grade III**	Zone 3 peri-sinusuidal peri-cellular fibrosis and peri-portal fibrosis with extensive or focal bridging
**Cirrhosis**	Cirrhosis
**Hepatocyte ballooning**	
**No ballooning**	No ballooning
**Grade I **	Sometimes, zone 3
**Grade II**	Evident, zone 3
**Grade III**	Symptomatic, more dominant in zone 3
**Lobar inflammation**	
**No change **	No change
**Grade I**	Diffuse neutrophils, monocytes at 1 or 2 points in a 20× microscopic field
**Grade II**	PMN with ballooning hepatocytes, chronic inflammation at 2 to 4 points in a 20× microscopic field
**Portal inflammation **	
**No change**	No change
**Grade I**	Mild, some portal areas
**Grade II**	Mild to moderate, most portal areas
**Grade III**	Moderate to severe, most portal areas

### 3.3. Statistical analysis

Statistical analyses were performed by SPSS® for Windows® ver 15 (SPSS Inc., Chicago, IL, USA). To test the differences between parametric and non-parametric variables in the two study groups, independent-sample Student's t test and Mann-Whitney U test were used. Categorical variables were analyzed by x(2) test. Kendall τ was calculated to evaluate the correlation betweefn continuous level of ALT and the categorical variable of hepatosteatosis grade. Moreover, to determine a better cut-off value for serum ALT to detect more advanced histopathologic features of patients with NAFLD, receiver operating characteristics (ROC) curve analysis was performed. The area under the curve (AUC) and the diagnostic values of each cut-off points including sensitivity and specificity were calculated. A p < 0.05 was considered statistically significant; a study power of 80% was assumed in analysis.

## 4. Results

4.1. Baseline characteristics

Among 147 studied patients with, 127 (86.4%) were male and 20 (13.6%) were female; they had a mean±SD age of 41.4±11.2 (range: 15-70) years. Other baseline characteristics including serum lipid profile and liver function test (LFT) of the patients are shown in Table 2. As it is shown, the mean±SD serum ALT, AST and Alk-P were 99.21±178.25, 54.89±32.41, and 185.04±184.17 U/L, respectively. All patients underwent liver biopsies, the results of which are shown in Table 3. The most frequent grade of hepatosteatosis was grade I in 66 patients (44.9%); the prevalence of grades II and III were 39.5% (58/147) and 15.6% (23/147), respectively. In addition, 103 (69.7%) patients with NAFLD patients did not have any evidence of fibrosis; liver cirrhosis was detected in five (3.4%) patients.

4.2 Characteristic and histopathologic features of patients with NAFLD regarding elevated ALT (> 35 U/L)

Regarding the cut-off value of 35 U/L for serum ALT level, the patients were divided into two groups; 22 patients had ALT < 35 U/L and 125 had ALT > 35 U/L. The baseline and clinical characteristics of patients with NAFLD stratified according to their serum ALT level are listed in Table 2. The mean ± SD serum total cholesterol level was significantly higher in those with elevated ALT (206.66 ± 51.10 vs. 176.85 ± 36.97 mg/dL, p = 0.014). The mean ± SD serum AST level was also significantly higher in patients with elevated ALT level (59.97 ± 31.92 vs. 25.14 ± 14.35 U/L, p < 0.001). Except for these two variables, the other baseline and laboratory characteristics of the patients were not significantly different between the two groups.

**Table 2:** Baseline and clinical characteristics of patients with NAFLD stratified by serum ALT level

**Variable**	**Total **(n = 147)	**Group of patients**		**P value**
		**ALT < 35 U/L **(n = 22)	**ALT > 35 U/L **(n = 125)	
**Age **(y) (Mean ± SD)	41.36 ± 11.18	42.71 ± 11.69	41.11 ± 11.11	0.548
**Gender **[No. (%)]				0.091
** Female**	20 (13.6%)	6 (27%)	14 (11.2%)	
** Male**	127 (86.4%)	16 (73%)	111 (88.8%)	
**BMI** (kg/m2) (Mean ± SD)	27.70 ± 3.80	26.77 ± 2.35	27.84 ± 3.96	0.329
**Fasting blood sugar **(mg/dL) (Mean ± SD)	102.02 ± 36.68	99.25 ± 13.68	102.44 ± 39.06	0.417
**Total cholesterol **(mg/dL) (Mean ± SD)	202.43 ± 50.33	176.85 ± 36.97	206.66 ± 51.10	0.014
**Triglyceride (mg/dL) **(Mean ± SD)	208.20 ± 132.15	206.00 ± 139.75	208.58 ± 131.43	0.470
**LDL-Cholesterol **(mg/dL) (Mean ± SD)	124.28 ± 42.39	122.13 ± 28.95	124.66 ± 44.47	0.833
**HDL-Cholesterol **(mg/dL) (Mean ± SD)	44.28 ± 19.67	39.00 ± 7.52	45.11 ± 20.86	0.236
**PTT **(s) (Mean ± SD)	12.49 ± 1.46	13.05 ± 0.61	12.40 ± 1.54	0.122
**Platelet count **(/L) (Mean ± SD)	343612 ± 479131	502000 ± 836990	325568 ± 425416	0.746
**AST **(U/L) (Mean ± SD)	54.89 ± 32.41	25.14 ± 14.35	59.97 ± 31.92	<0.001
**Alk-P **(U/L) (Mean ± SD)	185.04 ± 184.17	134.63 ± 60.84	193.09 ± 195.81	0.200

Patients' histopathologic findings are presented in Table 3. The most common grade of hepatosteatosis was grade I observed in 59.1% and 42.4% of patients with normal and elevated serum ALT level, respectively. There was no significant difference in the mean ± SD quantitative grade of hepatosteatosis between the two groups (1.50 ± 0.67 vs. 1.74 ± 0.73, p = 0.136). Advanced fibrosis (grade III and cirrhosis) was reported in 4.5% (1/22) and 5.6% (7/125) of those with normal and elevated ALT, respectively (p = 0.327). The mean ± SD grade of fibrosis was also similar in the two groups (0.32 ± 0.95 vs. 0.52 ± 0.94, p = 0.104). Other histopathologic findings were also not significantly different except for the mean grade of lobar inflammation (p = 0.045) (Table 3).

**Table 3:** Histopathologic characteristics of patients with NAFLD stratified by serum ALT level

**Finding**	**Total **(n = 147)	**Group of patients**		**P value**
		**ALT < 35 U/L **(n = 22)	**ALT > 35 U/L** (n = 125)	
**Hepatosteatosis**				0.324
** Grade I** [No. (%)]	66 (44.9%)	13 (59%)	53 (42.4%)	
** Grade II** [No. (%)]	58 (39.5%)	7 (32%)	51 (40.8%)	
** Grade III** [No. (%)]	23 (15.6%)	2 (9%)	21 (16.8%)	
** Grade** (Mean ± SD)	1.71 ± 0.72	1.50 ± 0.67	1.74 ± 0.73	0.136
**Fibrosis**				
** No fibrosis** [No. (%)]	103 (69.7%)	19 (87%)	84 (67.2%)	0.327
** Grade I** [No. (%)]	29 (20%)	1 (5%)	28 (22.4%)	
** Grade II **[No. (%)]	7 (4.8%)	1 (5%)	6 (4.8%)	
** Grade III** [No. (%)]	3 (2.1%)	—	3 (2.4%)	
** Cirrhosis** [No. (%)]	5 (3.4%)	1 (5%)	4 (3.2%)	
** Grade **(Mean ± SD)	0.49 ± 0.94	0.32 ± 0.95	0.52 ± 0.94	0.104
**Hepatocyte ballooning**				0.254
** No ballooning **[No. (%)]	127 (86.8%)	22 (100%)	105 (84%)	
** Grade I** [No. (%)]	12 (7.6%)	—	12 (9.6%)	
** Grade II** [No. (%)]	7 (4.9%)	—	7 (5.6%)	0.045
** Grade III** [No. (%)]	1 (0.7%)	—	1 (0.8%)	
** Grade** (Mean ± SD)	0.20 ± 0.54	0	0.23 ± 0.58	
**Lobar inflammation**				0.458
** No change** [No. (%)]	102 (69.7%)	17 (77.3%)	85 (68%)	
** Grade I** [No. (%)]	38 (26.1%)	5 (22.7%)	33 (26.4%)	
** Grade II** [No. (%)]	7(4.2%)	—	7 (5.6%)	
** Grade** (Mean ± SD)	0.35 ± 0.57	0.23 ± 0.43	0.38 ± 0.59	0.328
**Portal inflammation**				0.224
** No change** [No. (%)]	113 (77.1%)	19 (86.4%)	94 (75.2%)	
** Grade I** [No. (%)]	26 (17.4%)	2 (9.1%)	24 (19.2%)	
** Grade II** [No. (%)]	6 (4.2%)	—	6 (4.8%)	
** Grade III** [No. (%)]	2 (1.3%)	1 (4.5%)	1 (0.8%)	
** Grade** (Mean ± SD)	0.30 ± 0.61	0.23 ± 0.68	0.31 ± 0.60	0.282

4.3 Relationship between serum ALT level and histopathologic features of patients with NAFLD

Analysis was performed to evaluate the association between serum ALT level and histopathologic findings in patients with NAFLD. After exclusion of those with advanced fibrosis (No. = 8), a significant direct correlation was found between serum ALT level and grade of hepatosteatosis (p = 0.012). The mean ± SEM serum ALT level was also significantly higher in patients with NAFLD with > 33% hepatocytes involvement (126.99 ± 28.25 U/L in comparison to the those with mild hepatosteatosis (73.14 ± 5.98 U/L) (p = 0.020). Considering the cases of advanced fibrosis in analysis, the AST/ALT ratio was significantly higher in patients with NAFLD who suffered from advanced fibrosis (mean ± SD: 1.18 ± 0.46 vs. 0.75 ± 0.47, p = 0.012).

4.4 Receiver Operating Characteristics (ROC) curve analysis

**Figure 1:**
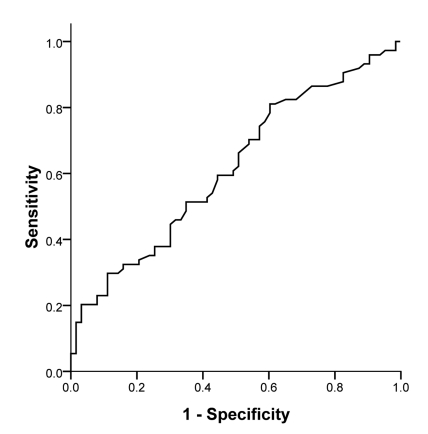
ROC curve analysis of serum ALT level to predict more than 33% hepatocytes involvement in hepatosteatosis of patients with NAFLD without advanced fibrosis. Area under curve [AUC] = 0.616, p = 0.020

**Figure 2:**
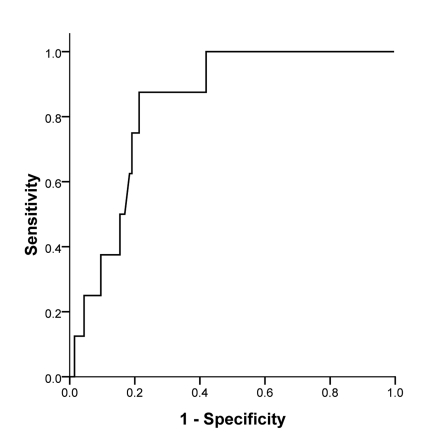
ROC curve analysis of AST/ALT ratio to predict advanced fibrosis in NAFLD patients. Area under curve (AUC)=0.836, P=0.001

To find a better cut-off value for serum ALT level to predict the histopathologic features of patients with NAFLD, ROC curve analysis was performed; it revealed that serum concentration of ALT could significantly predict the cases with > 33% hepatocytes involvement from the mild hepatosteatosis condition (AUC = 0.616, p = 0.020; Figure 1). In addition, a cut-off value of 58.5 U/L had a sensitivity of 66% and a specificity of 50% to detect patients with NAFLD with moderate to severe hepatosteatosis; a cut-off value of 77.5 U/L had a sensitivity of 51% and a specificity of 65% in these subjects. As illustrated in Figure 2, the AST/ALT ratio was also a statistically significant tool to predict advanced fibrosis (AUC = 0.836, p = 0.001). A cut-off value of 0.88 for the ratio had a sensitivity of 87.5% and a specificity of 79.7% to detect patients with severe fibrosis or/and cirrhosis.

## 5. Discussion

NAFLD which closely resembles alcohol-induced liver injury according to histological features is a clinico-histopathological entity that occurs in patients with little or no history of alcohol consumption. It has a wide histological spectrum ranging from fat accretion in hepatocytes without inflammation or fibrosis (simple hepatic steatosis) to hepatic steatosis with necro-inflammatory elements (steatohepatitis) that may or may not have associated fibrosis [17]. Presence of fibrosis and inflammation is termed "non-alcoholic steatohepatitis" (NASH), which may eventually progress to cirrhosis in up to 20% of patients [7]. ALT is found in the cytosol of hepatocytes where it transfers amino groups, while the major site for AST is mitochondria [18]. Amino group transfer from alanine to ketoglutarate is catalyzed by ALT. Even though, ALT is mostly specific to liver, it can be found in blood during muscle injuries or inflammation [19][20]. It has been suggested that elevated liver enzymes does not strongly correlate with the level of liver injury, fibrosis or inflammation [21]; however, it is a diagnostic evaluation hint.

The main objective of this study was to evaluate the pathologic findings in patients with NAFLD according to the ALT level (above vs. below 35 U/L). Our findings demonstrated that by considering the current cut-off value of 35 U/L, there is no significant difference between patients with NAFLD according to their pathologic findings except for ballooning of hepatocytes which is a minor finding compared to more frequent presentations such as fibrosis. This could be partially explained by the variability of ALT level in patients in NASH which has been confirmed by previous studies [22][23]. In the third National Health and Nutrition Examination Survey (NHANES), the variation in ALT was studied among two measurement results in the American people. About 30% of patients with firstly elevated ALT had normal values on the repeated testing. Likewise, our results concerning ALT in NAFLD subjects showed variability according to the histopathologic findings [22]. In another study, 29% of those with NAFLD with normal ALT had elevated ALT on repeated testing and 38% of those with elevated ALT had normal ALT at the next visit [23]. The high inconsistency was comparable in patients with simple steatosis and NASH. The within patient coefficient of variation for ALT in the second cohort was greater than that reported in NHANES (33.6 vs. 20.4) [22][23].

Previous studies have demonstrated that occurrence of significant fibrosis in NASH might be seen in patients with NAFLD who had a normal ALT value [14][24]. One option is that those were false reports due to the implementation of unsuitable ALT cut-off values. In a retrospective study of 51 patients with normal ALT levels, bridging fibrosis was seen in 12 and cirrhosis was found in six patients [14]. However, the cut-off value they used was 52 U⁄L for women and 75 U⁄L for men. On the other hand, the mean ALT level in patients with normal ALT was 40 U⁄L, which would be considered abnormal according to the current standard. Fracanzani et al. studied 63 patients with normal ALT and reported that normal ALT correlated with less severe steatosis and necro-inflammation; however, they could not find any correlations between the severity of fibrosis and ALT levels. They considered ALT values < 40 U/L as normal [25]. Another retrospective study performed by Kunde et al. on 233 obese women who underwent liver biopsies during bariatric surgery showed that patients with ALT < 19 U⁄L had less severe histopathologic findings. However, 23% and 5% of patients still had NASH and advanced fibrosis, respectively [26]. Wong et al. confirmed that ALT levels do not correlate well with metabolic and histological parameters in patients with NAFLD. Their results demonstrated that NASH and significant fibrosis can be found even among those with ALT below half of the upper normal limit[24]. Our results were also in concordance with these finding as we could not find any correlation between ALT levels and histopathologic or metabolic abnormalities in these patients. More recently, another multi-center study was conducted on 733 patients with NAFLD who had biopsy specimens; it showed that the presence of advanced fibrosis was not correlated with ALT levels [12]. It seem that using ALT level as a marker for severe NAFLD would result in considering high risk patients as mild cases as with normal ALT, there is still risk of progressive and severe hepatic disease.

Previous large population-based studies have shown that abnormal ALT levels (ALT > 43 U/L) are present in 2.8% of adult population which increases to 6.6% in class II or III obesity [27]. Using the same ALT cut-off value (43 U/L), another study performed by Kunde et al.[26] showed elevated ALT levels in 8.6% of their study population which consisted of women with class II or III obesity. In our study, we used a cut-off value of 30 U/L for ALT; according to this level, 125 (85%) of 147 patients had abnormal ALT levels. A comparison of our study to the mentioned studies is not possible because of the different target populations. Our study had several limitations considering the design. The most important of all was the small sample size which was due to the unacceptable nature of liver biopsy among patients. Secondly, the cross-sectional design of the study would be a limitation. Cohort studies with multiple biopsies during the course of disease while measuring ALT level in a periodic schedule would help to more precisely interpret the findings in correlation to ALT level. However, the invasiveness of liver biopsy procedure and ethical issues are the major limiting factor of such studies. We recommend larger multi-center cohorts with large sample sizes being followed with a unique protocol while their clinical, laboratory and histopathological data is being recorded. Such studies might be able to answer our questions considering the important issues about NAFLD course and severity. Altogether, our results underline the little contribution of ALT as an independent factor for detecting the severity of NAFLD. Therefore, using ALT levels as an indicator of severity might result in false reassurance of patients and physicians.
